# Epigenetic glycosylation of SARS-CoV-2 impact viral infection through DC&L-SIGN receptors

**DOI:** 10.1016/j.isci.2021.103426

**Published:** 2021-11-11

**Authors:** Lei Guo, Yan Liang, Heng Li, Huiwen Zheng, Zening Yang, Yanli Chen, Xin Zhao, Jing Li, Binxiang Li, Haijing Shi, Ming Sun, Longding Liu

**Affiliations:** 1 Institute of Medical Biology, Chinese Academy of Medical Science and Peking Union Medical College, Beijing, China; 2 Kunming Key Laboratory of Children Infection and Immunity, Yunnan Key Laboratory of Children's Major Disease Research, Yunnan Medical Center for Pediatric Diseases, Yunnan Institute of Pediatrics, Kunming Children's Hospital, Kunming, China

**Keywords:** Immunity, Virology, Cell biology, Cell, Glycomics

## Abstract

Glycosylation of severe acute respiratory syndrome coronavirus 2 (SARS-CoV-2) spike glycoprotein mediates viral entry and immune escape. While glycan site is determined by viral genetic code, glycosylation is completely dependent on host cell post-translational modification. Here, by producing SARS-CoV-2 virions from various host cell lines, viruses of different origins with diverse spike protein glycan patterns were revealed. Binding affinities to C-type lectin receptors (CLRs) DC&L-SIGN differed in the different glycan pattern virions. Although none of the CLRs supported viral productive infection, viral *trans*&cis-infection mediated by the CLRs were substantially changed among the different virions. Specifically, *trans*&cis-infection of virions with a high-mannose structure (Man_5_GlcNAc_2_) at the N1098 glycan site of the spike postfusion trimer were markedly enhanced. Considering L-SIGN co-expression with ACE2 on respiratory tract cells, our work underlines viral epigenetic glycosylation in authentic viral infection and highlights the attachment co-receptor role of DC&L-SIGN in SARS-CoV-2 infection and prevention.

## Highlights

• DC&L-SIGN are SARS-CoV-2 attachment co-receptor
• Viral spike (S) glycoprotein undergoes epigenetic modification during infection
• Epigenetic glycosylation affects viral in-*cis*&-*trans* infections through DC&L-SIGN
• High-mannose glycan at 1098 site of postfusion S trimer is vital for viral infection

## Graphical abstract

**Figure:**
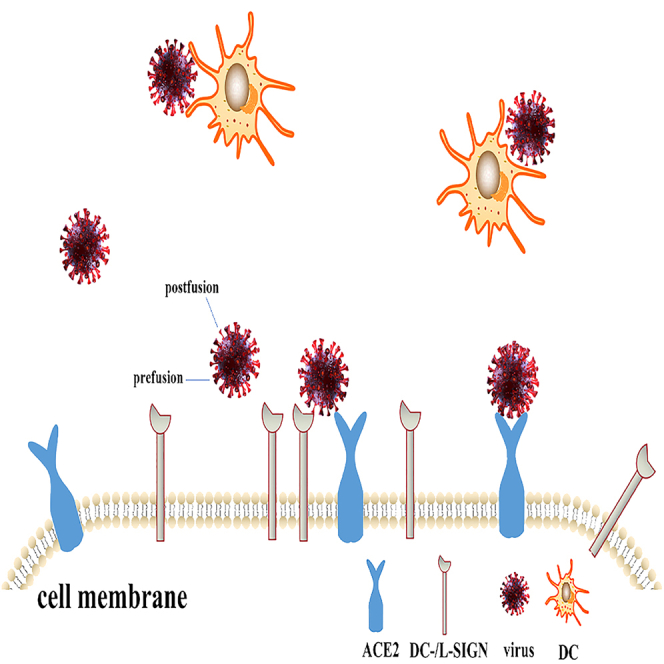

## Introduction

Over one and a half years after its outbreak, the coronavirus disease-2019 (COVID-19) pandemic caused by severe acute respiratory syndrome coronavirus 2 (SARS-CoV-2) remains ongoing. As an emerging highly infectious virus, what we know about viral pathogenesis is still little despite vaccine accessibility. The viral surface trimeric spike (S) glycoprotein binds to its receptor ACE2, and after binding the S trimer undergoes proteolytic cleavage and conformational change from the prefusion to postfusion form which triggers membrane fusion and delivers the viral genome into the cytosol to initiate replication (Lan et al., 2020). As a glycoprotein, S undergoes extensive glycosylation per protomer and glycans present on both prefusion and postfusion trimers of viral surface (Cai et al., 2020; Watanabe et al., 2020; Yao et al., 2020; Zhao et al., 2020). Heavy glycosylation of viral entry protein is considered a way of virus immune escape by forming a glycan shield (Watanabe et al., 2019). Moreover, glycan presented on S protein has been suggested to support S-ACE2 binding in conformation and mediate infection as ligands for lectin receptor binding (Casalino et al., 2020; Evans and Liu, 2021).

Glycosylation is a highly diverse process that produces abundant and highly complex glycans that are covalently attached to proteins, lipids, and even RNAs present on host cells and viruses (Flynn et al., 2021; Monteiro and Lepenies, 2017). Instead of template determination, glycosylation relies on a post-translational modification (PTM) process from ER to Golgi apparatus by glycosyltransferases and glycosidases with randomness. The host cell type, cell metabolic level, cell surrounding, and cell stimuli all have a strong influence on glycosylation of a specific glycoprotein (Butler and Spearman, 2014; Goh and Ng, 2018). Viruses hijack the host cell glycosylation machine for their glycoprotein (Monteiro and Lepenies, 2017); thus, the glycosylation profile of viruses may differ upon infection with all types of host cells under various physiological conditions. 22 N-linked glycosylations were assessed in S glycoprotein of SARS-CoV-2 (Watanabe et al., 2020). The 22 N-glycan sites remain highly conserved among the prototype virus and the emerging highly contagious viruses, including the alpha to delta variants. Only the T20N substitution in the gamma variant seems to acquire a new N-glycan site based on the N-glycosylation principle. The overall glycosylation states of the S protein produced from recombinant expression and viral infection were similar, however, composition of high-mannose structure differed (Watanabe et al., 2020; Yao et al., 2020).

High-mannose structure (Man_5-9_GlcNAc_2_) is a main ligand for two C-type lectin receptors (CLRs), DC-SIGN and L-SIGN (Guo et al., 2004; Mitchell et al., 2001). As pattern recognition receptors (PRRs), these CLRs sense glycans present on the surface of pathogens to activate antiviral immune responses. Moreover, some viruses, including HIV-1 (Geijtenbeek et al., 2000), Ebola virus (Alvarez et al., 2002), influenza virus (Wang et al., 2008), human cytomegalovirus (Halary et al., 2002), dengue virus (Tassaneetrithep et al., 2003), and SARS-CoV (Jeffers et al., 2004), have evolved to exploit CLRs as additional receptors for viral *trans*/cis infection. DC-SIGN is expressed by immature or mature DCs and specialized monocytes/macrophages (Khoo et al., 2008). L-SIGN, which shares 77% amino acid sequence identity with DC-SIGN, is present on endothelial cells in the liver, lymph nodes, lungs, and placenta (Khoo et al., 2008). L-SIGN has been found to bind SARS S glycoprotein and support viral infection as a functional viral receptor (Chan et al., 2006; Jeffers et al., 2004). In view of this, works have been performed to explore the role of DC/L-SIGN as a SARS-CoV-2 receptor. Studies by Amraie et al. and Soh et al. indicate that DC-SIGN and L-SIGN act as alternative entry receptors for SARS-CoV-2 infection using pseudotype virus system and authentic virions (Soh et al., 2020; Amraie et al., 2021). However, other works revealed that DC/L-SIGN alone does not allow direct cell infection and proliferation; in contrast, they mediate SARS-CoV-2 infection in the presence of ACE2 as co-receptor or auxiliary receptor (Lempp et al., 2021; Thepaut et al., 2021; Kondo et al., 2021). Monocyte-derived DCs (MDDCs) expressing DC-SIGN capture SARS-CoV-2 virions and promote virus transfer to infect ACE2+ Calu-3 cells (trans-infection) (Thepaut et al., 2021). A recent work found that overexpression of DC/L-SIGN in 293T cells enhances viral infection, suggesting the possibility of cis-infection by DC/L-SIGN (Lempp et al., 2021). Thus, DC- and L-SIGN play alternative receptors and/or co-receptors in SARS-CoV-2 infection. The discrepancy of DC-SIGN and L-SIGN receptors in mediating SARS-CoV-2 infection is possible owing to the heterogeneity of high-mannose binding ligands present on S glycoprotein of different produced virions. Thus, to explore epigenetic glycosylation of SARS-CoV-2 and the implication in viral infection, the virions were prepared from various host cell lines expressing ACE2 receptor and viral S glycan profiles and viral infections through these two CLRs were analyzed.

## Results

### DC- and L-SIGN bind to SARS-CoV-2 S protein via glycans but cannot directly support SARS-CoV-2 proliferation

**Figure 1:**
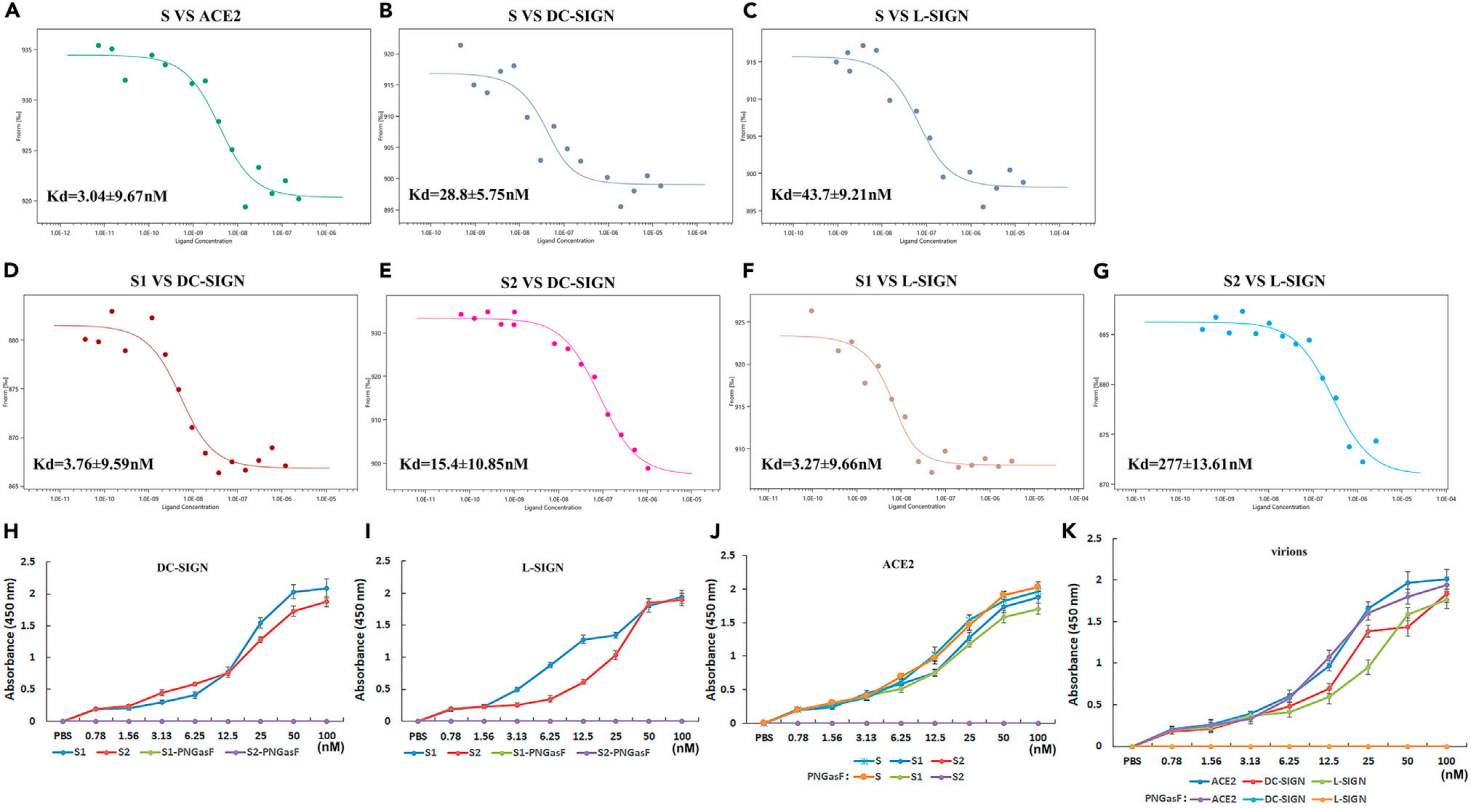
Binding of DC- and L-SIGN to SARS-CoV-2 and S glycoprotein (A–G) Interaction between recombinant S, S1, and S2 proteins and ACE2, DC-SIGN, and L-SIGN receptors, and the K_d_ values were measured by MST. (H–J) Binding of S, S1, and S2 proteins to DC-SIGN (H), L-SIGN (I), and ACE2 (J) receptors in the presence of PNGasF digestion by ELISA. (K) Binding of Vero cell origin virions to DC-SIGN, L-SIGN, and ACE2 receptors in the presence of PNGasF digestion by ELISA. The error bars represent the standard deviation from four repeats.

Affinities of S glycoprotein and DC/L-SIGN were measured by microscale thermophoresis (MST) assay and the results showed that both DC-SIGN and L-SIGN interact with recombinant S protein with a relatively lower affinity (K_d_, 28.8 nM, 43.7 nM, respectively) than that of the ACE2 receptor (K_d_, 3.04 nM) (Figures 1A, 1B, and 1C). Furthermore, both the S1 and S2 subunits of S protein interacted with DC-SIGN and L-SIGN with affinities varying from 3.27 to 277 nM (Figures 1D, 1E, 1F, and 1G). After treating viral proteins with PNGasF which specifically cleaves N-linked oligosaccharides, ELISA showed that the binding of recombinant S, S1, and S2 proteins to DC-SIGN and L-SIGN was eliminated, while the binding of ACE2 was retained (Figures 1H, 1I, and 1J). Moreover, the binding of SARS-CoV-2 virions produced from Vero cells infected with DC-SIGN and L-SIGN was also abolished when virions were pre-incubated with PNGasF (Figure 1K). Thus, the results suggest that SARS-CoV-2 virus binds to DC-SIGN and L-SIGN with S glycoprotein which is dependent on its N-glycans.

**Figure 2:**
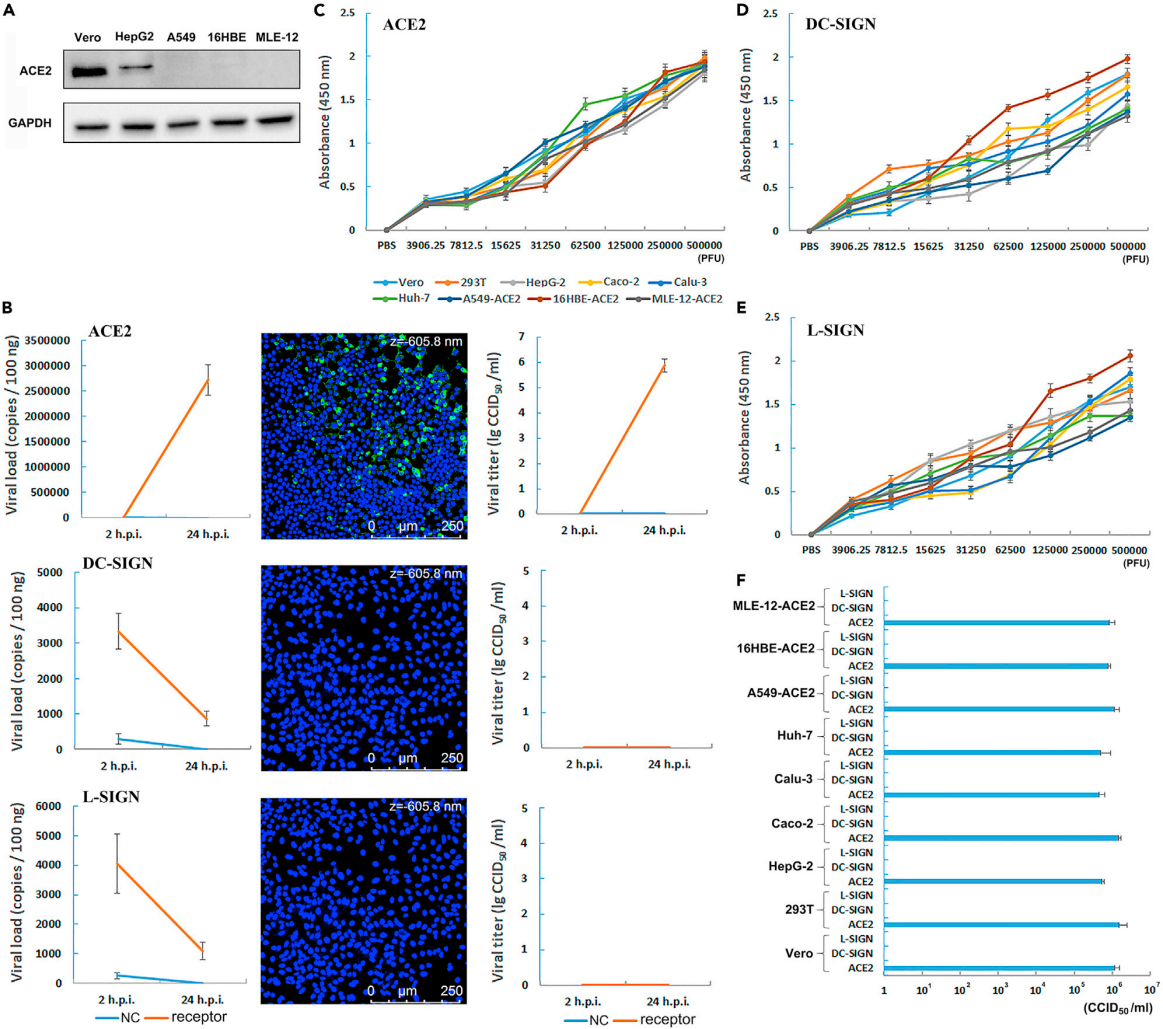
DC- and L-SIGN does not support productive infection of SARS-CoV-2 (A) Expression of ACE2 receptor in Vero, HepG-2, A549, 16HBE, and MLE-12 cells by western blot detection. Glyceraldehyde-3-phosphate dehydrogenase (GAPDH) was used as internal reference control. (B) SARS-CoV-2 infection, replication, and proliferation through MLE-12 cells that stably express ACE2, DC-SIGN, or L-SIGN receptor (from the top down). Cells transduced with empty lentiviral particles were used as a negative control (NC). Left panels, viral loads of the infected cells were determined based on the number of viral envelope gene RNA copies detected by qRT-PCR at the indicated h.p.i.; middle panels, virus-infected cells were visualized using anti-viral nucleocapsid protein antibody by confocal microscopy at 24 h.p.i.; right panels, viral titers from the culture supernatants of the infected cells were determined using a CCID_50_ assay at the indicated h.p.i.. (C–E) Binding of ACE2 (C), DC-SIGN (D), and L-SIGN (E) receptors to virions of different cell origins by ELISA. (F) Viral titers from the culture supernatants of infected ACE2-, DC-SIGN-, and L-SIGN-expressing MLE-12 cells upon infection with the virions of different cell origins at 24 h.p.i.. The error bars represent the standard deviation from four repeats.

A549 cells and MLE-12 cells that did not express human ACE2 receptor were used to stably express DC-SIGN or L-SIGN for SARS-CoV-2 infection (Figures 2A and S1). Although the virus bound to CLR expressing cells after incubation (2 h post-infection, 2 h.p.i.), DC-SIGN or L-SIGN does not support viral replication and proliferation based on the detection of viral loads, viral nucleocapsid protein expression, and viral titers at 24 h.p.i (Figures 2B and S2). Considering the glycosylation heterogenicity of S protein from different production systems, virions were propagated from various SARS-CoV-2 permissive host cell lines, including 293T, HepG-2, Caco-2, Calu-3, Huh-7, A549-ACE2, MLE-12-ACE2, and 16HBE-ACE2 cells (STAR Methods). The binding of the virions with various origins to ACE2, DC-SIGN, and L-SIGN receptors were evaluated with equal viral PFUs, and the results showed that all of the virions were able to bind the three receptors (Figures 2C, 2D, and 2E). While binding capacity of the different virions to ACE2 receptor tend to be consistent (Figure 2C), the binding affinities varied from 1.3 to 2.1 at peak values for DC-SIGN and L-SIGN receptors (Figures 2D and 2E). None of the SARS-CoV-2 viruses could productively infect DC/L-SIGN expressing MLE-12 cells compared with ACE2 expressing cells based on a viral proliferation assay (24 h.p.i. viral titer detection) (Figure 2F). The above results indicate that although the binding capacities of DC- and L-SIGN receptors differed from those of SARS-CoV-2 viruses of various origins, the two CLRs were not able to support productive viral infection like ACE2 receptor.

### DC- and L-SIGN enhance ACE2-mediated SARS-CoV-2 infection (cis-infection) and promotes virus transfer to permissive ACE2+ cells (trans-infection)

**Figure 3:**
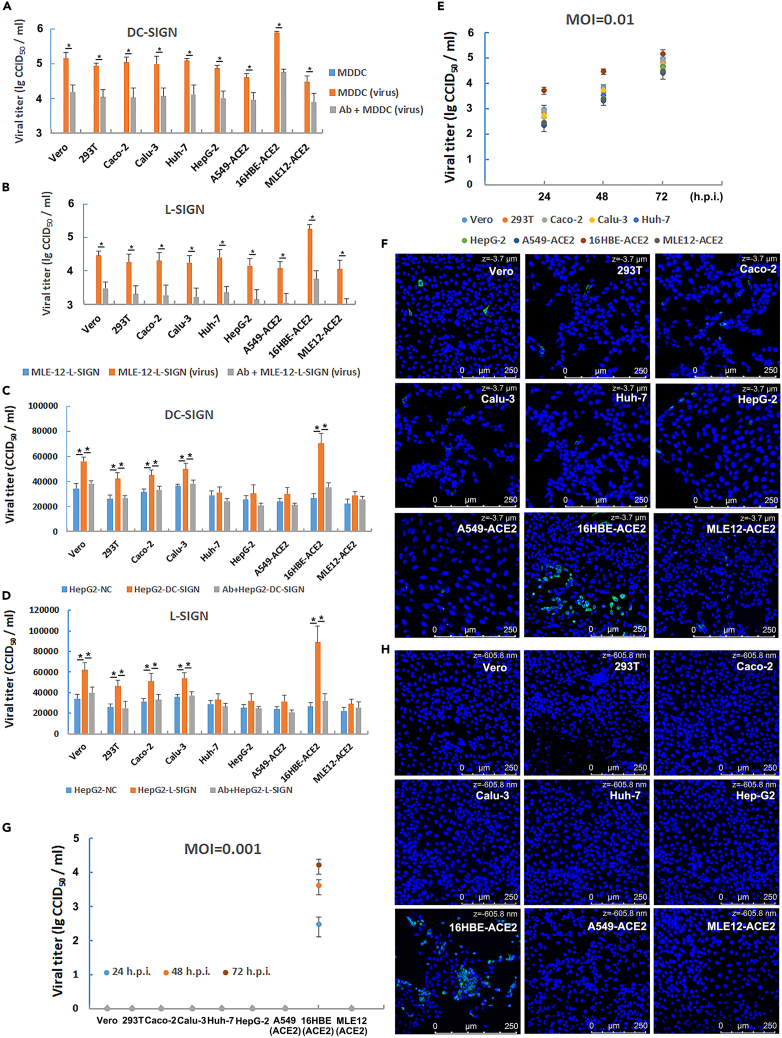
DC- and L-SIGN mediated SARS-CoV-2 *trans*/cis-infections (A and B) MDDCs (A) and MLE-12-L-SIGN cells (B) were first incubated with virions of different cell origins at a MOI = 1 for 1.5 h and then co-cultivated with Vero cells for 48 h after intensive washing. Viral titers of the different co-culture supernatants were determined by the CCID50 assay. MDDCs and MLE-12-L-SIGN cells (without virus incubation) were used as negative controls. For DC- and L-SIGN blockade, antibodies against the two receptors were used. (C and D) HepG-2 cells expressing DC-SIGN (C) and L-SIGN (D) were infected with viruses of different cell origins (MOI = 1), and viral titers were determined at 24 h.p.i. by CCID50 assay. HepG-2 cells transduced with empty lentiviral particles were used as a negative control (NC). For DC- and L-SIGN blockade, antibodies against the two receptors were used. (E–H) HepG-2 cells expressing L-SIGN were infected with viruses of different cell origins at a MOI of 0.01 (E and F) or 0.001 (G and H), viral titers were assessed at 24 –72 h.p.i. by the CCID50 assay (E and G), and virus-infected cells were visualized using anti-viral nucleocapsid protein antibody by confocal microscopy at 24 h.p.i. (F and H). The error bars represent the standard deviation from four repeats. ∗: P<0.05 based on Student's t-test.

MDDCs that expressed DC-SIGN (Figure S3) and MLE-12-L-SIGN cells were challenged with the virions (MOI = 1) for 1.5 h, and after intensive washing, they were co-cultivated with permissive Vero cells. The results showed that both CLRs can mediate trans-infection of SARS-CoV-2 viruses from MDDCs and MLE-12-L-SIGN cells to Vero cells (Figures 3A and 3B). Among the different virions, viruses from 16HBE-ACE2 cells most potently facilitated trans-infection via DC- and L-SIGN. Antibodies against DC/L-SIGN remarkably reduced DC/L-SIGN-mediated trans-infection further confirming the attachment receptor role of DC- and L-SIGN in SARS-CoV-2 trans-infection.

For investigating co-receptor role of DC/L-SIGN in ACE2 dependent cis-infection, HepG-2 cells that express low endogenous levels of ACE2 (Figure 2A) together with relatively inferior SARS-CoV-2 proliferation (STAR Methods) were chosen to conduct cis-infection assays. We first confirmed that lentiviral transduction of HepG-2 cells with DC/L-SIGN did not affect the endogenous expression of ACE2 (Figure S4) and then infected the transduced HepG-2 cells with SARS-CoV-2 viruses of different origins (MOI = 1). Viral titers were significantly increased in virions from Vero, 293T, Caco-2, Calu-3, and 16HBE-ACE2 cells at 24 h.p.i. in the presence of DC/L-SIGN, and proliferation was mostly enhanced in virions from 16HBE-ACE2 cells (an approximately 3-fold increase) (Figures 3C and 3D). Anti-DC/L-SIGN antibodies reduced DC/L-SIGN-mediated cis-infection, further supporting the co-receptor role of DC- and L-SIGN in SARS-CoV-2 cis-infection (Figures 3C and 3D). The strong potency of DC- and L-SIGN in facilitating viral infection of virions from 16HBE-ACE2 cells suggests the significance of co-receptor role of CLRs in low-level viral load infection. Considering that L-SIGN co-expresses with ACE2 on human lung tissue cells (Amraie et al., 2021; Chan et al., 2006) and expression of ACE2 *in vivo* is generally low in single-cell RNA sequencing datasets (Hou et al., 2020; Zou et al., 2020), the co-receptor role of L-SIGN may have straightforward meaning in natural respiratory infection occuring upon a tiny virus content. Thus, we further examined the cis-infection role of L-SIGN at a low multiple of infection (MOI). We first tested viral infections at a MOI of 0.01, and the results showed that viral proliferation of the 16HBE-ACE2 virions was much higher than that of the other virions at 24–72 h.p.i (Figures 3E and 3F). Moreover, virus from 16HBE-ACE2 cells could also infect and proliferate to a considerable amount of virions at 72 h.p.i. at a much lower MOI (0.001), while the other viruses were not able to proliferate (Figures 3G and 3H). Together, the results suggested that DC- and L-SIGN can facilitate SARS-CoV-2 cis-infection with the ACE2 receptor, and viruses produced from 16HBE-ACE2 cells most efficiently take advantage of DC- and L-SIGN as an infection attachment co-receptor.

### SARS-CoV-2 S protein glycosylation under host epigenetic control and high-mannose (Man_5_GlcNAc_2_) glycosylation N1098 site of S protein contributes to enhanced viral binding to L-SIGN receptor and facilitates viral infection

**Figure 4:**
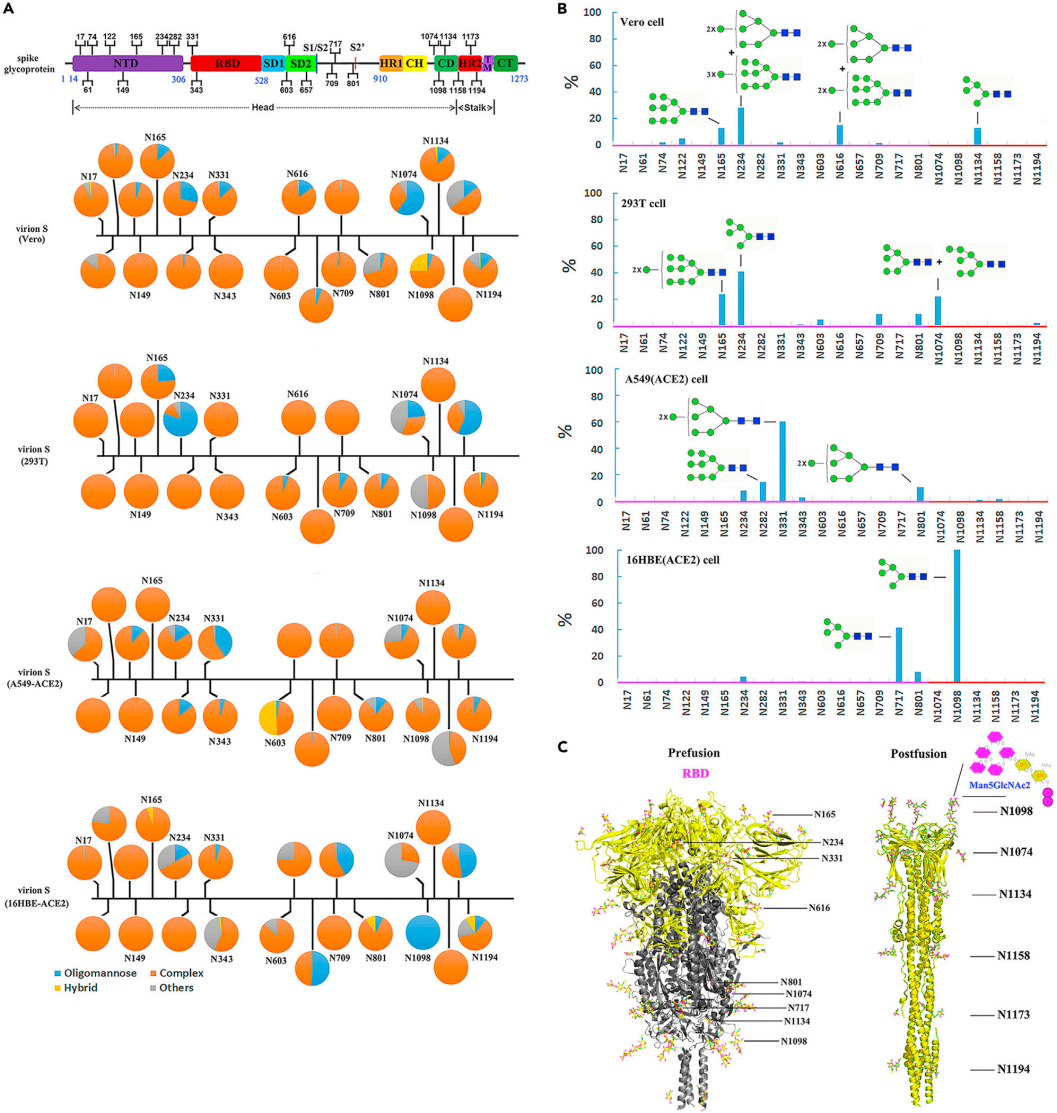
Site-specific N-link glycosylation of SARS-CoV-2 S glycoprotein (A) S proteins from Vero, 293T, A549-ACE2, and 16HBE-ACE2 cell origin virions were analyzed by LC-MS/MS, and compositions of different types of glycans (oligomannose, hybrid, complex, and others) at 22 N-link glycan sites are presented in pie charts. Upper panel, schematic representation of N-link glycan sites and structural domains in S glycoprotein. (B) Different types and proportions of high-mannose glycans (Man_5-12_GlcNAc_2_) at 22 N-link glycan sites of S glycoprotein from Vero, 293T, A549-ACE2, and 16HBE-ACE2 cell origin virions. (C) N-link glycosylation sites of the SARS-CoV-2 S trimer in the prefusion (PDB 6XR8) and postfusion conformations (PDB 6XRA). A representative glycan presented at each site was modeled manually on the N-linked carbohydrate attachment sites and marked in red by PyMOL software.

Site-specific N-glycan analysis of native S protein in virions from Vero, 293T, A549-ACE2, and 16HBE-ACE2 cells was conducted by liquid chromatography-tandem mass spectrometry (LC-MS/MS) analysis. Glycan compositions of total 22 N-glycosylation sites in S protein were revealed (Figure 4A and Table S1). The overall S protein N-glycan modifications of the tested viruses were mainly highly processed complex glycan types (Figure 4A). Distribution of underprocessed oligomannose and hybrid types were scattered in S1, S2, and also stalk subunits of S protein with relatively lower levels of oligomannose glycosylation at N61, N122, N234, N603, N709, N717, and N801 compared with the recombinant S trimer protein (Watanabe et al., 2020; Zhao et al., 2020). Among the S proteins of virions of different origins, glycosylation processing levels and types at each N-glycan site differed from each other, even though the S protein from virions of Vero cell origin presented an inconsistent glycosylation state with another native Vero origin S protein (Yao et al., 2020), highlighting that SARS-CoV-2 S glycosylation was essentially affected by host glycan epigenetic modification.

High-mannose glycans are binding ligands of DC- and L-SIGN receptors, and structures of Man_5-12_GlcNAc_2_ were observed across the S protein of the 4 virions (Figure 4B). High-mannose glycans (Man_5,8,9,11,12_GlcNAc_2_) were concentrated at N165, N234, N282, N331, and N616 of the S1 subunit, with abundances varying from 10% to 60%. Three N-glycan sites (N165, N234, and N331) are close to the ACE2 receptor-binding domain (RBD) in steric (Figure 4C), which supports the notion that high-mannose structures at these sites help to stabilize receptor binding via RBD (Casalino et al., 2020). High-mannose structures (Man_5-9_GlcNAc_2_) were detected at N717, N801, N1074, N1098, and N1134 of the S2 subunit (Figure 4B). The overall frequency of high-mannose glycans in S2 subunits was relatively lower than that of S1 subunits in virions from Vero, 293T, and A549 (ACE2) cells, while a high proportion of Man_5_GlcNAc_2_ structures was detected at N1098 (100% occupation) and N717 (>40%) in virions from 16HBE (ACE2) cells (Figure 4B). Notably, high-mannose structures were mainly distributed at the S2 subunit of virions from 16HBE (ACE2) cells, in contrast to Vero, 293T, and A549 (ACE2) cell origin virions, in which high-mannose glycans dominated the S1 subunit.

**Figure 5:**
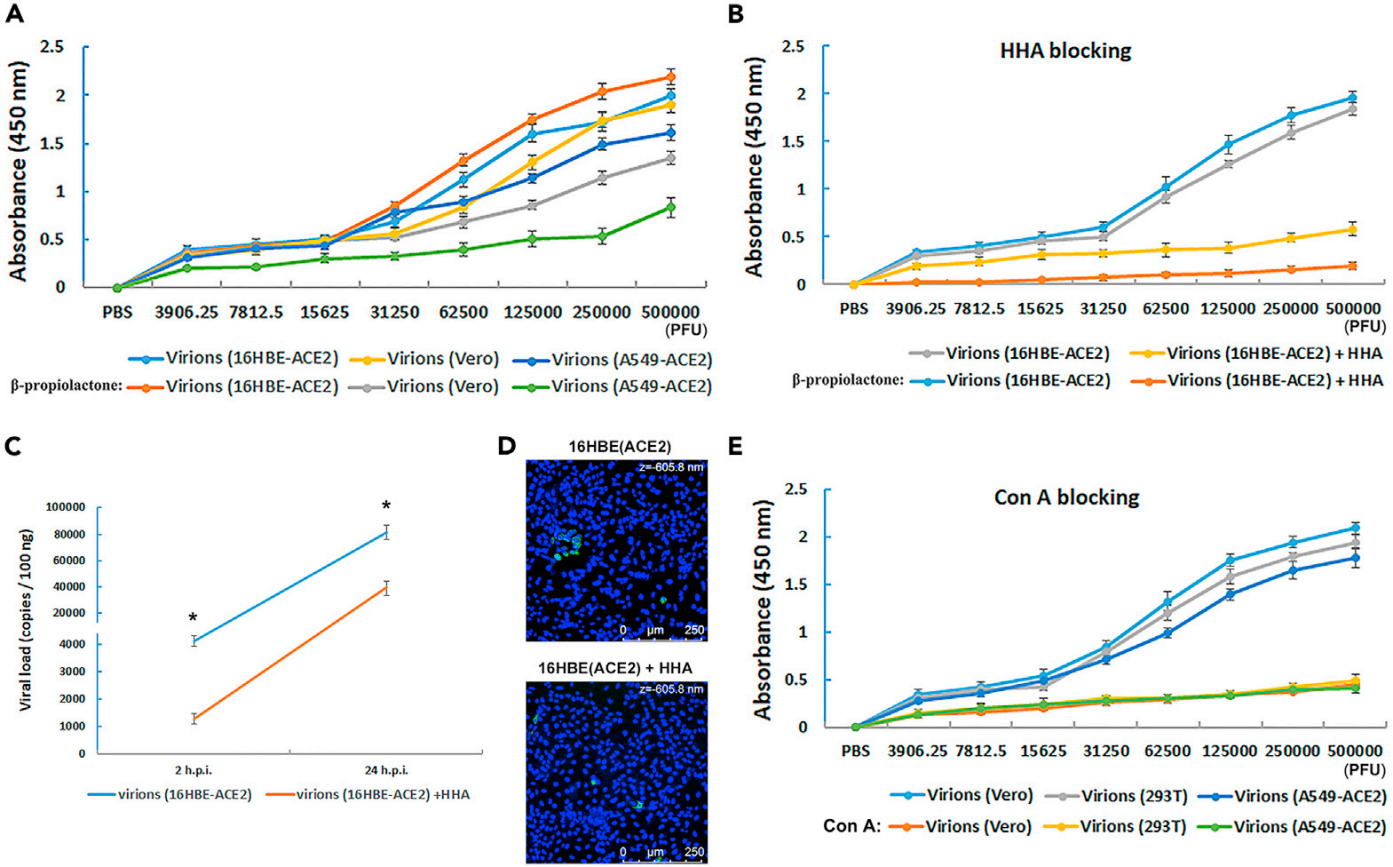
SARS-CoV-2 virions bind to DC/L-SIGN receptor primarily through high-mannose glycans (A) Virions from Vero, A549-ACE2, and 16HBE cells were inactivated with or without β-propiolactone and then bound to L-SIGN receptor by ELISA assay. (B) 16HBE (ACE2) cell origin virions which were inactivated with or without β-propiolactone were treated with lectin HHA (2 μg mL^−1^) before binding to L-SIGN receptor in ELISA assay. (C and D) 16HBE (ACE2) cell origin virions were pre-treated with or without lectin HHA and then infected HepG2 (L-SIGN) cells (MOI = 1). Viral loads of the infected cells were determined based on the number of viral envelope gene RNA copies detected by qRT-PCR at the indicated h.p.i. (C); The virus-infected cells were visualized using anti-viral nucleocapsid protein antibody by confocal microscopy at 24 h.p.i. (D). (E) Virions from Vero, A549-ACE2, and 293T cells were pre-treated with or without lectin Con A (0.5 μM) and then bound to DC-SIGN receptor by ELISA assay. The error bars represent the standard deviation from four repeats. ∗: P<0.05 based on Student's t-test.

Apparently, high-mannose glycans present on the top of the S1 head, such as N165, N234, and N331, instead of N717 and N1098, which are located close to the stalk domain of the S protein, are more easy to access for DC- and L-SIGN binding based on the S protein prefusion structure (Figure 4C). However, virions from 16HBE(ACE2) cells with primary S2 high-mannose glycosylation showed the strongest binding and employment of DC- and L-SIGN as co-receptor for infection than the other S1 high-mannose predominant virions (Figure 3). In authentic SARS-CoV-2 virus, there are prefusion and postfusion conformations of the S trimer protein present on the virion surface (Ke et al., 2020; Klein et al., 2020; Turonova et al., 2020). The postfusion conformation is a trimeric hairpin structure formed by conformational changes in the prefusion trimer after receptor binding and proteolytic cleavage (Figure 4C) (Ke et al., 2020; Klein et al., 2020; Turonova et al., 2020). The postfusion trimer is also found on the surface of mature virions and is supposed to be derived from the unstable prefusion trimer, which is cleaved by host proteinases when the progeny virus buds through the cell membrane (Cai et al., 2020; Xia et al., 2020). The proportion of the postfusion form of S trimer in mature virions varies from rare (Vero cell origin) to medium (Calu3 cell origin) to predominant (A549-ACE2 cell origin) levels (Ke et al., 2020; Klein et al., 2020; Turonova et al., 2020). N-link glycan decorations were assessed in postfusion structures at the N1098, N1074, N1134, N1158, N1173, and N1194 sites, among which N1098 was present on the outward apical site of the rod architecture (Figures 4B and 4C) (Cai et al., 2020; Yao et al., 2020). Based on the postfusion structure, the fully occupied Man_5_GlcNAc_2_ high-mannose glycan at the N1098 site in the postfusion S trimer of 16HBE (ACE2) cell origin virions may play a vital role in binding to DC- and L-SIGN receptors and facilitating DC- and L-SIGN-mediated infection. To further prove this, the viruses were treated with β-propiolactone to produce postfusion trimer dominant virions (Gao et al., 2020b; Liu et al., 2020), and the binding ability to the L-SIGN receptor was tested via ELISA. The results showed that after β-propiolactone treatment, the binding affinities of virions from Vero and A549 (ACE2) cells were remarkably decreased, while the binding affinity of virions from 16HBE(ACE2) cells was not impaired and slightly enhanced (Figure 5A), suggesting that the virions from 16HBE (ACE2) cells bind to L-SIGN receptor primarily through postfusion S trimer. To test whether the virions from 16HBE (ACE2) cells bind to DC/L-SIGN receptor through high-mannose structure at the N1098 site of the postfusion S trimer, lectin of *Hippeastrum Hybrid* (Amaryllis) (HHA), which specifically bind terminal α-1,3- and/or α-1,6-mannose of high-mannose oligosaccharides (Man_5_GlcNAc_2_) but not hybrid oligosaccharides (Kalu et al., 1990; Mitchell et al., 2017), was introduced to conduct Man_5_GlcNAc_2_ glycan-binding blocking assay. After pre-incubation of the virions from 16HBE (ACE2) cells with HHA, binding of the virions to L-SIGN receptor was markedly diminished in ELISA assay (Figure 5B). Binding to L-SIGN receptor of the β-propiolactone inactivated 16HBE (ACE2) origin virions was almost eliminated when pre-treatment the virions with HHA (Figure 5B). Pre-treatment 16HBE (ACE2) origin virions with HHA also restrained viral infection and proliferation in HepG2 (L-SIGN) cells based on detections of viral loads and viral nucleocapsid protein expression (Figures 5C and 5D). Thus, the above data support that SARS-CoV-2 virus from 16HBE (ACE2) cells binds to DC- and L-SIGN receptors via its high-mannose glycan present at the N1098 site in the postfusion S trimer to enhance viral infection in *cis* and *trans*. On the other hand, the binding of virions from Vero, 293T, and A549 (ACE2) cells to DC- and L-SIGN receptors is mainly dependent on high-mannose glycans present in the S1 subunit of the prefusion trimer (Figure 4D). Pre-treatment of virions from Vero, 293T, and A549 (ACE2) cells with lectin Concanavalin A, which binds to α-linked mannose oligosaccharide, markedly reduced the virions bound to DC-SIGN receptor in ELISA assay (Figure 5E), proving that these virions binding to DC/L-SIGN receptor primarily through N-linked high-mannose glycans despite that DC-SIGN also recognizes Fuc on Lex and LDNF epitopes located at the complex type N331- and N343-linked glycans of SARS-CoV-2 (Lenza et al., 2020). The relatively low abundance of the S1 high-mannose glycans in Vero and 293T cell origin virions (Figure 4B) contributed to their inferior binding to DC- and L-SIGN receptors and the following induction of viral *trans*/cis-infection in comparison with the 16HBE(ACE2) cell origin virions. Considerable high-mannose glycans (60%) at the N331 site of the S1 trimer were observed in A549(ACE2) cell origin virions (Figure 4B), while their binding affinity was not superior to those of virions from Vero and 293T cells (Figures 2D, 2E, and 4D), which is likely due to overexpression of ACE2 inducing the primary postfusion trimer on the viral surface of virions from A549-ACE2 cells (Klein et al., 2020). Together, the above data indicate that the binding of SARS-CoV-2 virus to DC&L-SIGN receptors via high-mannose glycan depends on the glycosylation site and glycan abundance, and high proportion of high-mannose glycan at N1098 site of S trimer is critical for viral binding to DC/L-SIGN receptor and facilitates infection.

## Discussion

CLRs DC- and L-SIGN were found to bind to SARS-CoV-2 S glycoprotein (Gao et al., 2020a; Amraie et al., 2021; Kondo et al., 2021; Lu et al., 2021; Thepaut et al., 2021), while their function in inducing the proinflammatory response was revealed (Lu et al., 2021), their role as viral infection receptors was contradictory. Unlike ACE2, which recognizes the S protein RBD, DC- and L-SIGN bind specific viral protein PTM molecules, high-mannose glycans. Because glycosylation is not a template-driven process but rather depends on host PTM machine, the glycan profile of viral glycoproteins can be diverse and complex. Our results underline host glycosylation epigenetic modification on SARS-CoV-2 S protein glycan composition. Consequently, different glycan compositions of viruses from various host cells will contribute to distinctly exploring DC- and L-SIGN receptors for infection, which is well documented in our work. Our work revealed that a high-mannose structure (Man_5_GlcNAc_2_) occupied the N1098 site of the postfusion S trimer and had a strong potency to facilitate viral *trans*/cis-infection via DC- and L-SIGN as co-receptors, indicating that a glycan decorated postfusion structure present on the surface of SARS-CoV-2 virus (Yao et al., 2020) is not redundant and may have a substantial function in receptor binding and mediating viral infection. Our data cannot completely exclude the possibility of DC- and L-SIGN as a direct entry receptor for SARS-CoV-2 infection, since there may be virions in authentic infection which possess highly occupied high-mannose structures at N-glycan sites close to the RBD of prefusion trimer that are able to bind the receptors more tightly and trigger conformational changes in the S protein.

The glycan composition of SARS-CoV-2 virions would be manifold during *in vivo* infection. Fortunately, all 22 N-glycan sites of the S protein remain highly conserved among all the predominant SARS-CoV-2 variants, indicating selection pressure to preserve these sites. Recombinant expression of the S trimer *in vitro* displayed more oligomannose-type glycans than the native S trimer (Watanabe et al., 2020; Zhao et al., 2020) which is advantageous for inducing more protective high-mannose antibodies. Based on the role of the glycosylation postfusion S trimer in mediating viral infection, using the full-length S protein as an immunogen may be better than using only the S1 or RBD subunit, the former of which could induce antibodies against the postfusion S trimer.

Overall, our work revealed that DC- and L-SIGN play an attachment co-receptor role in facilitating SARS-CoV-2 infection in *cis* and *trans* via distinct high-mannose binding patterns. The virus may use DC-SIGN, which is expressed on DC cells, for dissemination, similar to trans-infection in HIV. Moreover, considering that L-SIGN is co-expressed with ACE2 on human lung tissue cells (Amraie et al., 2021; Chan et al., 2006) and that the expression of L-SIGN is on more than 60% of SARS-CoV-2-infected pulmonary cells (Lu et al., 2021), L-SIGN may help the virion bind to ACE2 receptor by anchoring the virus to the cell membrane of ACE2-and L-SIGN-expressing cells. This is particularly important in view of the low expression of ACE2 in the respiratory tract.

### Limitations of the study

To confirm that the high-mannose N1098 virus binds to DC&L-SIGN receptors and enhances infection through the glycan site, N1098 glycan site mutation virus propagated from 16HBE (ACE2) cells can directly demonstrate. Owing to the strict prohibition of virus mutation operation under biosafety regulation of Chinese Academy of Medical Science, we are not allowed to carry out the mutation virus experiment. Considering that changing the S protein expression system, whether it is a different virus infection system or a recombinant expression system, will lead to changes in the glycan type and proportion at each glycosylation site of the S protein; it is difficult to reproduce the unique S protein glycosylation of the 16HBE (ACE2) cell origin virions and demonstrate the critical role of high-mannose N1098 site using pseudo-typed virus mutation experiment. Thus, based on the glycan analysis and lectin blocking assay, our study strongly indicated but not demonstrated that the N1098 site with high-mannose glycan of S trimer is a critical binding site of DC/L-SIGN receptor for facilitating SARS-CoV-2 infection.

## STAR★Methods

### Key resources table

**Table:** 

REAGENT or RESOURCE	SOURCE	IDENTIFIER
**Antibodies**		

Mouse monoclonal anti-SARS-CoV-2 spike S2	Zheng et al., (2020)	Cat# 2B2, RRID:AB_2833139
Mouse monoclonal anti-DC-SIGN	R and D Systems	Cat# MAB161, RRID:AB_357808
Mouse monoclonal anti-DC-SIGNR	R and D Systems	Cat# MAB162, RRID:AB_2244985
Mouse monoclonal anti-SARS-CoV-2 spike S1	Sino Biological	Cat# 40150-MM02, RRID:AB_2860459
Mouse monoclonal anti-ACE2	Sino Biological	Cat# 10108-MM36
Rabbit polyclonal anti-mouse IgG	Abcam	Cat# ab6728, RRID:AB_955440
Rabbit monoclonal anti-ACE2	Abcam	Cat# ab108209, RRID:AB_10862654
Rabbit monoclonal anti-SARS-CoV-2 spike	Sino Biological	Cat# 40150-R007, RRID:AB_2827979
Goat polyclonal anti-mouse IgG Alexa 488	Abcam	Cat# ab150113, RRID:AB_2576208
Goat polyclonal anti-rabbit IgG Alexa 488	Abcam	Cat# ab150081, RRID:AB_2734747
Goat polyclonal anti-mouse IgG Alexa 647	Abcam	Cat# ab150119, RRID:AB_2811129
Donkey polyclonal anti-rabbit IgG Alexa 647	Abcam	Cat# ab150075, RRID:AB_2752244
Rabbit polyclonal anti-ACE2	Abcam	Cat# ab15348, RRID:AB_301861
Rabbit polyclonal anti-GAPDH	Abcam	Cat# ab9485, RRID:AB_307275
Rabbit polyclonal anti-beta actin	Abcam	Cat# ab8227, RRID:AB_2305186
Mouse monoclonal anti-SARS spike	Abcam	Cat# ab273433, RRID:AB_2891068

**Bacterial and virus strains**		

SARS-CoV-2-KMS1/2020 GenBanl:MT226610.1	Zheng et al. (2020)	N/A

**Biological samples**		

Health adult peripheral blood mononuclear cells	Institute of Medical Biology, Chinese Academy of Medical Science	http://www.imbcams.ac.cn

**Chemicals, peptides, and recombinant proteins**		

Dulbecco’s modified Eagle medium (DMEM)	ThermoFisher	Cat# 11965092
Eagle’s minimum essential medium	ThermoFisher	Cat# 21010046
Ham's F-12 Nutrient Mixture	ThermoFisher	Cat# 11765054
Fetal bovine serum	ThermoFisher	Cat# 10099141
Penicillin and streptomycin	ThermoFisher	Cat# 15140148
Hippeastrum hybrid lectin	Vector	Cat# L-1380-5
Concanavalin A	Sigma	Cat# L7647
Recombinant human GM-CSF	Novoprotein	Cat# GMP-CC79
Recombinant human IL-4	Novoprotein	Cat# GMP-CD03
HEPES	ThermoFisher	Cat#c 15630080
EDTA	ThermoFisher	Cat# AM9260G
Tween-20	ThermoFisher	Cat# 003005
Recombinant SARS-CoV-2 S trimer	Novoprotein	Cat# DRA49
Recombinant SARS-CoV-2 S1	Novoprotein	Cat# DRA47
Recombinant SARS-CoV-2 S2	Novoprotein	Cat# DRA48
Recombinant ACE2	Novoprotein	Cat# C419
Recombinant DC-SIGN	R and D Systems	Cat# 161-DC
Recombinant DC-SIGNR	R and D Systems	Cat# 162-D2
Paraformaldehyde	Solarbio	Cat# P1110
β-propiolactone	Solarbio	Cat# P9620
PNGase F	New England Biolabs	Cat# P0709L
PBS	ThermoFisher	Cat# 10010002
bovine serum albumin	Solarbio	Cat# A8010
TRIzol Reagent	Tiangen	Cat# DP424
4’,6-diamidino-2-phenylindole (DAPI)	Beyotime	Cat# C1002
RIPA lysis buffer	Solarbio	Cat# R0010
SDS-PAGE	Solarbio	Cat# P1213
SuperSignal West Pico PLUS Chemiluminescent Substrate	ThermoFisher	Cat# 34580
Pierce Crosslink Magnetic IP/Co-IP Kit	ThermoFisher	Cat# 88805
DL-dithiothreitol	Sigma	Cat# R0861
Iodoacetamide	Sigma	Cat# I10188
Trypsin	Promega	Cat# VA5111
Asp-N	Promega	Cat# V1621
Glu-C	Promega	Cat# V1651
Chymotrypsin	Promega	Cat# V1062

**Critical commercial assays**		

Ficoll density gradient centrifugation	Solarbio	Cat# P8680
EasySep™ Human Monocyte Enrichment Kit	Stemcell	Cat# 19059
Tetramethylbenzidine ELISA Substrate	Solarbio	Cat# PR1210
One Step PrimeScriptTM RT-PCR Kit	Takara	Cat# RR064A

**Experimental models: Cell lines**		

African green monkey: Vero E6 cells	ATCC	CRL-1568
Human: 293T cells	ATCC	CRL-3216
Human: Huh-7 cells	ATCC	PTA-4583
Human: HepG-2	ATCC	HB-8065
Human: Caco-2 cells	ATCC	HTB-37
Human: Calu-3 cells	ATCC	HTB-55
Human: RPMI-2650 cells	ATCC	CCL-30
Human: A549 cells	ATCC	CCL-185
Mouse: MLE-12	ATCC	CRL-2110
Human: 16HBE	NICR	CL-249

**Oligonucleotides**		

Primer: viral envelope gene Forward: ACAGGTACGTTAATAGTTAATAGCGT	This paper	N/A
Primer: viral envelope gene Reverse: ATATTGCAGCAGTACGCACACA	This paper	N/A
Prober: viral envelope gene: ACACTAGCCATCCTTACTGCGCTTCG	This paper	N/A

**Software and algorithms**		

Byonic	Protein Metrics	http://proteinmetrics.com/byos
Protein Data Bamk	RCSB PDB	http://www1.rcsb.org
PyMOL	Schrödinger	http://www.pumol.org
Excel	Windows Office 365	http:www.office.com
LAS X	Leica	https://www.leica-microsystems.com/products/microscope-software/p/leica-las-x-ls/
CFX Manager Software	Bio-Rad	https://www.bio-rad.com/en-kr/sku/1845000-cfx-manager-software?ID=1845000

### Resource availability

#### Lead contact

Further information and requests for resources and reagents should be directed to and will be fulfilled by the lead contact, Longding Liu (liuld@imbcams.com.cn).

#### Materials availability

This study did not generate new unique reagents.

### Experimental model and subject details

#### Cell lines

Vero (African green monkey, kidney,), 293T (human, kidney), Huh-7 (human, liver), HepG-2 (human, liver), Caco-2 (human, colon), Calu-3 (human, lung), A549 (human, lung), 16HBE (human, lung), RPMI-2650 (human, nose), and MLE-12 (mouse, lung) cells were obtained from Chinese National Infrastructure of Cell Line Resource (NICR) and American Type Culture Collection (ATCC). Vero, 293T, and Huh-7 cells were incubated in Dulbecco’s modified Eagle medium (DMEM) (ThermoFisher Scientific, USA). HepG-2, Caco-2, Calu-3, and RPMI-2650 cells were incubated in Eagle’s minimum essential medium (ThermoFisher Scientific, USA). A549, 16HBE, and MLE-12 cells were incubated in DMEM/F-12 medium with nutrient mix (ThermoFisher Scientific, USA). All cell lines were incubated in the above media containing 10% fetal bovine serum (FBS), 100 U ml^-1^ penicillin, and 100 μg ml^-1^ streptomycin (ThermoFisher Scientific, USA) at 37°C and 5% CO_2_. Cells stably expressing human ACE2, DC-SIGN, or L-SIGN receptors were generated by retroviral transduction and puromycin-based selection (ACE2 and L-SIGN) and hygromycin-based selection (DC-SIGN). Peripheral blood mononuclear cells (PBMCs) were isolated by Ficoll density gradient centrifugation (Solarbio, China) from blood samples of healthy human donors at Institute of Medical Biology (IMB), Chinese Academy of Medical Science (CAMS) under informed consent and IRB/IEC approval. To generate monocyte-derived dendritic cells (MDDCs), CD14+ monocytes were purified using anti-human CD14 antibody-labeled magnetic beads and EasySep Magent (Stemcell, Canada) from PBMCs. Differentiation to immature MDDCs was achieved by incubation of CD14+ monocytes at 37°C with 5% CO_2_ for 7 days and activation with GM-CSF (1000 U mL^-1^) and IL-4 (500 U mL^-1^) (Novoprotein, China) every second day.

#### SARS-CoV-2 viruses

The viral strain SARS-CoV-2-KMS1/2020 (GenBank accession number: MT226610.1) was isolated from sputum collected from a COVID-19 patient by IMB, CAMS, and propagated and titered on Vero cells in DMEM supplemented with 2% (vol/vol) FCS, 150 U ml^-1^ penicillin, and 150 μg ml^-1^ streptomycin at 37°C with 5% CO_2_. The stock virus was frozen at -80°C and prepared for following experiments. All experiments involving infectious viruses were performed with prior approval by the Institutional Biosafety Committee of IMB and conducted in the biosafety level (BSL)-3 laboratory of the National High Level Biosafety Laboratory Animal Center (Kunming).

For SARS-CoV-2 virus production in various cell lines, the cell monolayers (70-80% confluent) were incubated with SARS-CoV-2 virus from Vero cells at a MOI of 1 for 1 h, then washed with PBS twice and cultured at 37°C with 5% CO_2_. At 24 h.p.i., the cell culture media were harvested for virus titration by 50% tissue culture infective dose (TCID_50_) assay or plaque assay on Vero cells. Progeny viruses from the different cell lines were consecutively propagated in their host cells for 3 generations under the same experimental procedure before use in subsequent experiments. After 3 generation passages, the viruses of different origins were sequenced (Figure S5), and their titers were assessed. Among them, Vero, Caco-2, and Calu-3 cells support efficient SARS-CoV-2 proliferation (titer, > 10^7^ ml^-1^), while 293T (titer, 10^5^ ml^-1^), Huh-7 (titer, 10^5.5^ ml^-1^), HepG-2 (titer, 10^4^ ml^-1^), A549 (titer, 10^2^ ml^-1^), and 16HBE (titer, < 10^2^ ml^-1^) cells support moderate to limited viral proliferation. Viral titers were under detection of MLE-12 and RPMI-2650 cells. A549, 16HBE, and MLE-12 cells were conducted to stable express ACE2 by lentiviral transduction, and the corresponding A549-ACE2, 16HBE-ACE2, and MLE12-ACE2 cells efficiently supported SARS-CoV-2 virus with titers > 10^7^ ml^-1^.

### Method details

#### Affinity measurement by microscale thermophoresis assay

The binding affinities of the ligand to the receptor were measured using the MST method on a Monolith NT.115 instrument (NanoTemper Technologies, Germany). The ligand, purified recombinant S, S1, and S2 proteins were fluorescently labeled with the Monolith NT Protein Labeling Kit RED (NanoTemper, catalogue no: MO-L003). The fluorescently labeled ligands were kept at a constant concentration of 4 nM. The unlabeled ACE2, DC-SIGN and L-SIGN proteins were added at concentrations ranging from 6 nM to 20 μM in 0.01 M HEPES buffer containing 0.15 M NaCl, 3 nM EDTA, 0.005% Surfactant P20 and 0.05% Tween-20. The ligand and receptor were mixed and loaded into MonolithTM standard-treated capillaries, and thermophoresis was performed on a Monolith NT.115 instrument according to the manufacturer’s recommendations. The Kd (dissociation constant) was determined using NanoTemper analysis software.

#### ELISA assay

Recombinant SARS-CoV-2 S trimer (DRA49), S1 protein (DRA47), S2 protein (DRA48), and human ACE2 protein (C419) were produced by a mammalian expression system (HEK293 cell) purchased from Novoprotein (Shanghai, China). Recombinant DC-SIGN/CD209 (161-DC) and L-SIGN/CD299 proteins (162-D2) were produced by mouse myeloma cell line purchased from R&D Systems (Minneapolis, USA). Lectins of *Hippeastrum Hybrid* (Amaryllis) (HHA) (Vector, USA) and Concanavalin A (Sigma, USA) were used as 2 μg ml^-1^ and 0.5 μM, respectively. The virus was either inactivated with 3% paraformaldehyde (vol/vol) or 0.05% β-propiolactone (vol/vol) for 48 h at 4°C. The enzymatic digestion of recombinant protein and virions by PNGase F (P0709L, New England Biolabs, UK) followed the protocols recommended by the vender.

A 96-well High-Binding Flat-Bottom Microplate (Corning, USA) was incubated with different concentrations of recombinant proteins or virions at 4°C overnight. Proteins were 2-fold serial dilutions starting from 100 nM in protein coating assays. Virions were 2-fold serial dilutions starting from 5×10^5^ PFU in virion coating assays. PBS was used as a negative control. After removal of the coating supernatant, the plates were washed 4 times with ice-cold PBS containing 0.1% Tween 20 (PBST) and blocked with 0.2 ml of PBS containing 5% bovine serum albumin (BSA) at room temperature for 2 h. After washing with ice-cold PBST, the plates were incubated with anti-ACE2 (10108-MM36, SinoBiological, China), anti-DC-SIGN (R&D Systems Cat# MAB161), anti-L-SIGN (R and D Systems Cat# MAB162), and anti-viral spike (40150-MM02, SinoBiological) antibodies at 4°C overnight. After washing with PBST, the plate was incubated at room temperature for 1 h with horseradish peroxidase-conjugated rabbit anti-mouse IgG antibody (ab6728, Abcam, UK). The plate was then incubated with 200 μl of tetramethylbenzidine (TMB) ELISA Substrate (Solarbio, China) for 15 min at room temperature. Finally, the reaction was stopped with 100 μl of 0.5 M H_2_SO_4_. The absorbance was determined at 450 nm using a plate reader.

#### Viral load and viral titer

RNA was isolated from the cell lysates using TRIzol Reagent (Tiangen Biotechnologies, China) according to the manufacturer’s guidelines. The RNA concentration of each sample was determined by measuring the absorbance at 260 nm using a NanoDrop 2000. In all, 100 ng of total RNA was reverse transcribed and amplified using a One Step PrimeScript^TM^ RT-PCR Kit (Takara Biotechnologies, China) on a 7500 Fast Real-Time PCR system (Applied Biosystems, USA). To determine viral loads, primers and a viral envelope (E) gene probe were used: 5'-ACAGGTACGTTAATAGTTAATAGCGT-3' (forward), 5'-ATATTGCAGCAGTACGCACACA-3' (reverse), and 5'-(FAM)- ACACTAGCCATCCTTACTGCGCTTCG-(TAMRA)-3' (probe). The E gene of the virus was cloned into the pMD18-T vector, which was used to create a standard curve by 10-fold serial dilution. Viral copy numbers were normalized to the mass of the original cell samples and calculated based on the standard curve described above.

For viral titer, cell culture supernatants from the infected cells were centrifuged at 5 000 r/min for 10 min at 4°C, after which the supernatants were collected. Viral titers were determined using CCID50 titers by serial titration of viruses in Vero cells. Titers were calculated based on the method of Reed and Muench. A plaque assay was used to quantify infectious viruses from cell culture supernatants. Briefly, the harvested supernatants were serially diluted 10-fold and inoculated into confluent Vero cells in 6-well plates. After 2 h of incubation to allow virus attachment, the wells were gently washed with PBS, covered with 2 mL of semisolid DMEM containing 2% FBS and 0.3 Gum Tragacanth, and inverted in a 37°C CO_2_ incubator for 72 h. Next, the cells were fixed with 2 mL of 4% paraformaldehyde at 15°C to 25°C for 30 min, and the 1% agarose was removed. The monolayer of cells was stained with a crystal violet staining solution for 15 min and washed with distilled water. Finally, visible plaques were counted and the plaque-forming units (PFU mL^-1^) were calculated with the virus titer formula, in which the virus titer equals the number of plaques × (1 mL) × dilution factor.

#### Trans-infection and cis-infection assay

MDDCs and MLE-12-L-SIGN cells stably expressing DC-SIGN or L-SIGN were seeded at 5×10^4^ cells per well in 24-well plates. The next day, cells were inoculated with SARS-CoV-2 viruses at a MOI of 1 at 37°C for 1.5 h. For antibody-mediated inhibition, cells were preincubated with 10 μg ml^-1^ anti-DC-SIGN or anti-L-SIGN antibodies for 45 min. After inoculation, cells were washed 5 times with complete medium and 1×10^5^ Vero cells were added per well and incubated for 24 h at 37°C for trans-infection. Viral titers from the coculture supernatants were assessed at the endpoint. For cis-infection assay, 16HBE cells stably expressing DC-SIGN or L-SIGN were infected with SARS-CoV-2 virus at an MOI of 1, 0.01, or 0.001. After inoculation for 1.5 h, the cells were washed 3 times and then cultivated for 72 h. At 24, 48, and 72 h.p.i., the cells were subjected to immunofluorescence detection, and the culture supernatants were harvested for viral titer determination.

#### Immunofluorescence

Cells were fixed and blocked with 5% BSA, and then incubated overnight at 4°C with rabbit anti-human ACE2 monoclonal antibody (ab108209; Abcam, UK), mouse anti-DC-SIGN monoclonal antibody (MAB161, R&D Systems, USA), mouse anti-L-SIGN monoclonal antibody (MAB162, R&D Systems, USA), or rabbit anti-SARS-CoV-2 spike monoclonal antibody (40150-R007, Sino Biological, China), depending on the experimental design. The cells were then incubated with Alexa Fluor 488-conjugated goat anti-mouse IgG (ab150113; Abcam, UK), 488-conjugated goat anti-rabbit IgG (ab150081; Abcam, UK), Alexa Fluor 647-conjugated goat anti-mouse IgG (ab150119; Abcam, UK) or Alexa Fluor 647-conjugated goat anti-rabbit IgG (ab150075; Abcam, UK) for 1 h at room temperature. Nuclei were counterstained with 4’,6-diamidino-2-phenylindole (DAPI, Beyotime, China). Images were obtained with a Leica TCS SP8 laser confocal microscope (Leica Microsystems, Germany).

#### Western blotting

Protein extracts were prepared from different cells using RIPA lysis buffer (Solarbio, China). Equal amounts of protein were separated using sodium dodecyl sulfate-polyacrylamide gel electrophoresis (SDS-PAGE) and transferred to polyvinylidene fluoride (PVDF) membranes. Membranes were stained with rabbit anti-human ACE2 polyclonal antibody (ab15348; Abcam, UK), rabbit anti-GAPDH polyclonal antibody (ab9485, Abcam, UK), or rabbit anti-β-actin polyclonal antibody (ab8227, Abcam, UK). Proteins were detected using SuperSignal West Pico PLUS Chemiluminescent Substrate (ThermoFisher Scientific, USA).

#### Sample and material preparations for LC-MS/MS

Enough virus samples for LC-MS/MS proliferated from Vero, 293T, A549-ACE2, and 16HBE-ACE2 cells in T75 culture flasks. The cell supernatants were cleared from cell debris by centrifugation at 3500 g for 30 min, and SARS-CoV-2 virions were immunoprecipitated using an anti-SARS spike antibody (ab273433, Abcam, UK) with a Pierce Crosslink Magnetic IP/Co-IP Kit (88805, ThermoFisher Scientific, USA). The purified virions were resolved by SDS-PAGE, and the bands corresponding to the S protein and S1 S2 subunits were cut and analyzed by LC-MS/MS.

DL-dithiothreitol (DTT), iodoacetamide (IAA), formic acid (FA), acetonitrile (ACN), and methanol were purchased from Sigma (MO, USA). Proteases were purchased from Promega (WI, USA). Ultrapure water was prepared from a Millipore purification system (MA, USA). An Ultimate 3000 system coupled with an Orbitrap Fusion™ Lumos™ Tribrid™ Mass Spectrometer with an ESI nanospray source was obtained from ThermoFisher (Scientific, USA).

#### Protein digestion

The SARS-CoV-2 spike proteins were digested by trypsin&Asp-N, trypsin&Glu-C, and chymotrypsin. Gel slices were cut into 1 mm3 cubes, and the gel cubes were transferred to a 1.5 mL microcentrifuge tube. The tube was centrifuged for 1-2 sec to spin the gel slices to the bottom of the tube. Then, 50 μL of 30 mmol/L K3Fe(CN)6: 100 mmol/L Na_2_S_2_O_3_ = 1:1 (vol/vol) was added, followed by washing until the brown disappeared and removing the supernatant immediately. Next, 200 μL of water was adeed to stop the reaction for 10 min, the supernatant was removed, and 100 μL of 100 mm NH_4_HCO_3_ was added. The solution was allowed to stand for 20 min, the supernatant was removed. 500 μL of acetonitrile was added and the solution was incubated for 10 min. The gel pieces should become opaque and stick together.

The acetonitrile was removed using a pipettor with a clean pipette tip, and the gel slices were rehydrated in 10 mM DTT/50 mM ammonium bicarbonate, followed by the addition of enough solution to completely cover the gel slices. After incubation at 56°C for 1 hour, the supernatant was removed, and 500 μL of acetonitrile was added, followed by incubation for 10 min. The gel pieces should become opaque and stick together. The acetonitrile was removed using a pipettor with a clean pipette tip. Then, 50 mM IAA and 50 mM ammonium bicarbonate were added to completely cover the gel slices. After incubation for 1 h at room temperature in the dark, the IAA/ammonium bicarbonate was removed using a clean pipette tip, 500 μL of acetonitrile was added, and the solution was incubated for 10 min. The gel pieces should become opaque and stick together. The acetonitrile solution was removed, and just enough enzyme digestion solution was added to cover the gel slices. The gel pieces were incubated on ice for 45 min, and more digestion solution was added if all of the initial solution was absorbed by the gel pieces. Then, 5-20 μL of enzyme digestion solution was added to keep the gel pieces wet during enzymatic digestion. Following incubation overnight at 37°C, a pipettor and a clean pipette tip were used to recover the supernatant and transfer it into a fresh 1.5 mL microcentrifuge tube. Then, 100 μL of 50 mM ammonium bicarbonate/acetonitrile solution (1:2, vol/vol) was added to cover the gel slices, followed by incubation for 1 hour at 37°C. The solution was extracted and transferred to a 1.5 mL microcentrifuge tube, and then, the extracted peptides were lyophilized to near dryness.

#### Nano LC-MS/MS analysis

Nano LC-MS/MS analysis was conducted by BiotechPack Scientific (Beijing, China). Before analysis, the peptides were reconstituted in 10 μL of 0.1% formic acid. LC-MS/MS was performed on an Orbitrap Fusion™ Lumos™ Tribrid™ mass spectrometer coupled with an Ultimate 3000 System. For each sample, 5 μL of volume was loaded onto a C18 PepMap100 trap column (300 μm×5 mm) and eluted on a Thermo Acclaim PepMap RPLC analytical column (150 μm×15 cm). A 120 min gradient for each single-shot analysis was performed as follows: 4-10% B in 5 min, 10-22% B in 80 min, 22-40% B in 25 min, 40-95% B in 5 min, and 95-95% B in 5 min (A=0.1% formic acid in water, B=0.1% formic acid in 90% acetonitrile). The flow rate was 0.6 μL min^-1^. The data-dependent mode was operated for the mass spectrometer, with a full MS scan (350- 1550 m/z) and a 3 s cycle time. The MS spectra were acquired at a resolution of 70,000 with an automatic gain control (AGC) target value of 3×10^6^ ions or a maximum integration time of 40 ms. High-energy collision dissociation (HCD) with the energy set at 27 NCE was used to perform peptide fragmentation. The MS/MS spectra were acquired in the top 15 or 20 most intense precursors at a resolution of 17500 with an AGC target value of 1×10^5^ ions or a maximum integration time of 60 ms.

#### MS Data Analysis

The raw MS files were analyzed and searched against the target protein database based on the species of the samples using Byonic software (V3.6, Protein Metrics, USA). The mass tolerance was set to 20 ppm and 0.02 Da for the precursor and the fragment ions, respectively, with up to two missed cleavages allowed. Carbamidomethyl (+57.021 Da) (C) was used as a fixed modification, while oxidation (+15.995 Da) (M) and N-glycan 309 mammalian no sodium.txt@NGlycan were used as variable modifications. The results of protein identification were filtered with the criteria of a mass tolerance less than 10 ppm for peptides and a false positive rate less than 1% at the protein level. Only peptides with high confidence were chosen for downstream protein identification analysis. N-linked glycans were categorized into 5 major classes according to the composition detected. HexNAc(2)Hex(1-12) were classified as oligomannose type with HexNAc(2)Hex(5-12) classified as high-mannose type; HexNAc(3)Hex(5-9)Fuc(0-1)NeuAc(0-1) were classified as hybrid type; and HexNAc(>3) and HexNAc(3)Hex(3-4) were classified as complex type. The rest of the types including HexNAc(1) and HexNAc(2) were classified as others.

#### Model building of S trimer

Model buildings were based on the cryogenic electron microscopy (Cryo-EM) structure of the SARS-CoV-2 S prefusion trimer (Protein Data Bank, PDB: 6XR8) and postfusion trimer (PDB: 6XRA) and performed manually using PyMOL software (V2.5, Schrödinger, USA). A representative glycan presented at each site was modeled on the N-linked carbohydrate attachment sites.

### Quantification and statistical analysis

Quantified data were analyzed statistically with Excel for Windows (Office 365, Microsoft, USA). The data obtained from all experiments are presented as mean±SD, and P<0.05 using Student’s t-test indicated statistical significance.

## Data Availability

All data reported in this paper will be shared by the lead contact upon request. Any additional information required to reanalyze the data reported in this paper is available from the lead contact upon request.

## Supplementalinformation

Document S1. Figures S1–S6

Table S1. N-glycosylation of S protein at 22 N-glycan sites of the SARS-CoV-2 viruses

## References

[bib1] Alvarez C.P., Lasala F., Carrillo J., Muniz O., Corbi A.L., Delgado R. (2002). C-type lectins DC-SIGN and L-SIGN mediate cellular entry by Ebola virus in cis and in trans. J. Virol..

[bib2] Amraie R., Napoleon M.A., Yin W., Berrigan J., Suder E., Zhao G., Olejnik J., Gummuluru S., Muhlberger E., Chitalia V. (2021). CD209L/L-SIGN and CD209/DC-SIGN act as receptors for SARS-CoV-2. ACS Cent. Sci..

[bib3] Butler M., Spearman M. (2014). The choice of mammalian cell host and possibilities for glycosylation engineering. Curr.Opin.Biotechnol..

[bib4] Cai Y., Zhang J., Xiao T., Peng H., Sterling S.M., Walsh R.M., Rawson S., Rits-Volloch S., Chen B. (2020). Distinct conformational states of SARS-CoV-2 spike protein. Science.

[bib5] Casalino L., Gaieb Z., Goldsmith J.A., Hjorth C.K., Dommer A.C., Harbison A.M., Fogarty C.A., Barros E.P., Taylor B.C., McLellan J.S. (2020). Beyond shielding: the roles of glycans in the SARS-CoV-2 spike protein. ACS Cent. Sci..

[bib6] Chan V.S., Chan K.Y., Chen Y., Poon L.L., Cheung A.N., Zheng B., Chan K.H., Mak W., Ngan H.Y., Xu X. (2006). Homozygous L-SIGN (CLEC4M) plays a protective role in SARS coronavirus infection. Nat. Genet..

[bib7] Evans J.P., Liu S.L. (2021). Role of host factors in SARS-CoV-2 entry. J. Biol. Chem..

[bib8] Flynn R.A., Pedram K., Malaker S.A., Batista P.J., Smith B.A.H., Johnson A.G., George B.M., Majzoub K., Villalta P.W., Carette J.E. (2021). Small RNAs are modified with N-glycans and displayed on the surface of living cells. Cell.

[bib9] Gao C., Zeng J., Jia N., Stavenhagen K., Matsumoto Y., Zhang H., Li J., Hume A.J., Muhlberger E., van Die I. (2020). SARS-CoV-2 spike protein interacts with multiple innate immune receptors. bioRxiv.

[bib10] Gao Q., Bao L., Mao H., Wang L., Xu K., Yang M., Li Y., Zhu L., Wang N., Lv Z. (2020). Development of an inactivated vaccine candidate for SARS-CoV-2. Science.

[bib11] Geijtenbeek T.B., Kwon D.S., Torensma R., van Vliet S.J., van Duijnhoven G.C., Middel J., Cornelissen I.L., Nottet H.S., KewalRamani V.N., Littman D.R. (2000). DC-SIGN, a dendritic cell-specific HIV-1-binding protein that enhances trans-infection of T cells. Cell.

[bib12] Goh J.B., Ng S.K. (2018). Impact of host cell line choice on glycan profile. Crit. Rev. Biotechnol..

[bib13] Guo Y., Feinberg H., Conroy E., Mitchell D.A., Alvarez R., Blixt O., Taylor M.E., Weis W.I., Drickamer K. (2004). Structural basis for distinct ligand-binding and targeting properties of the receptors DC-SIGN and DC-SIGNR. Nat. Struct. Mol. Biol..

[bib14] Halary F., Amara A., Lortat-Jacob H., Messerle M., Delaunay T., Houles C., Fieschi F., Arenzana-Seisdedos F., Moreau J.F., Dechanet-Merville J. (2002). Human cytomegalovirus binding to DC-SIGN is required for dendritic cell infection and target cell trans-infection. Immunity.

[bib15] Hou Y.J., Okuda K., Edwards C.E., Martinez D.R., Asakura T., Dinnon K.H., Kato T., Lee R.E., Yount B.L., Mascenik T.M. (2020). SARS-CoV-2 reverse genetics reveals a variable infection gradient in the respiratory tract. Cell.

[bib16] Jeffers S.A., Tusell S.M., Gillim-Ross L., Hemmila E.M., Achenbach J.E., Babcock G.J., Thomas W.D., Thackray L.B., Young M.D., Mason R.J. (2004). CD209L (L-SIGN) is a receptor for severe acute respiratory syndrome coronavirus. Proc. Natl. Acad. Sci. U S A.

[bib17] Kalu H., Damme E.J.V., Peumans W.J., Glodstein I.J. (1990). Carbohydrate-binding specificity of the daffodil (Narcissus pseudonarcissus) and amaryllis (Hippeastrum hybr.) bulb lectins. Arch. Biochem.Biophys..

[bib18] Ke Z., Oton J., Qu K., Cortese M., Zila V., McKeane L., Nakane T., Zivanov J., Neufeldt C.J., Cerikan B. (2020). Structures and distributions of SARS-CoV-2 spike proteins on intact virions. Nature.

[bib19] Khoo U.S., Chan K.Y., Chan V.S., Lin C.L. (2008). DC-SIGN and L-SIGN: the SIGNs for infection. J. Mol. Med..

[bib20] Klein S., Cortese M., Winter S.L., Wachsmuth-Melm M., Neufeldt C.J., Cerikan B., Stanifer M.L., Boulant S., Bartenschlager R., Chlanda P. (2020). SARS-CoV-2 structure and replication characterized by in situ cryo-electron tomography. Nat. Commun..

[bib21] Kondo Y., Larabee J.L., Gao L., Shi H., Shao B., Hoover C.M., McDaniel J.M., Ho Y.C., Silasi-Mansat R., Archer-Hartmann S.A. (2021). L-SIGN is a receptor on liver sinusoidal endothelial cells for SARS-CoV-2 virus. JCI Insight.

[bib22] Lan J., Ge J., Yu J., Shan S., Zhou H., Fan S., Zhang Q., Shi X., Wang Q., Zhang L. (2020). Structure of the SARS-CoV-2 spike receptor-binding domain bound to the ACE2 receptor. Nature.

[bib23] Lempp F.A., Soriaga L., Montiel-Ruiz M., Benigni F., Noack J., Park Y.J., Bianchi S., Walls A.C., Bowen J.E., Zhou J. (2021). Lectins enhance SARS-CoV-2 infection and influence neutralizing antibodies. Nature.

[bib24] Lenza M.P., Oyenarte I., Diercks T., Quintana J.I., Gimeno A., Coelho H., Diniz A., Peccati F., Delgado S., Bosch A. (2020). Structural characterization of N-linked glycans in the receptor binding domain of the SARS-CoV-2 spike protein and their interactions with human lectins. Angew. Chem. Int. Ed. Engl..

[bib26] Liu C., Mendonca L., Yang Y., Gao Y., Shen C., Liu J., Ni T., Ju B., Liu C., Tang X. (2020). The architecture of inactivated SARS-CoV-2 with postfusion spikes revealed by cryo-EM and cryo-ET. Structure.

[bib27] Lu Q., Liu J., Zhao S., Gomez Castro M.F., Laurent-Rolle M., Dong J., Ran X., Damani-Yokota P., Tang H., Karakousi T. (2021). SARS-CoV-2 exacerbates proinflammatory responses in myeloid cells through C-type lectin receptors and Tweety family member 2. Immunity.

[bib28] Mitchell D.A., Fadden A.J., Drickamer K. (2001). A novel mechanism of carbohydrate recognition by the C-type lectins DC-SIGN and DC-SIGNR.Subunit organization and binding to multivalent ligands. J. Biol. Chem..

[bib29] Mitchell C.A., Ramessar K., O'Keefe B.R. (2017). Antiviral lectins: selective inhibitors of viral entry. Antivir. Res..

[bib30] Monteiro J.T., Lepenies B. (2017). Myeloid C-type lectin receptors in viral recognition and antiviral immunity. Viruses.

[bib31] Soh W.T., Liu Y., Makayama E.E., Omo C., Torii S., Nakagami H., Matsuura Y., Shioda T., Arase H. (2020). The N-terminal domain of spike glycoprotein mediates SARS-CoV-2 infection by associating with L-SIGN and DC-SIGN. bioRxiv.

[bib32] Tassaneetrithep B., Burgess T.H., Granelli-Piperno A., Trumpfheller C., Finke J., Sun W., Eller M.A., Pattanapanyasat K., Sarasombath S., Birx D.L. (2003). DC-SIGN (CD209) mediates dengue virus infection of human dendritic cells. J. Exp. Med..

[bib33] Thepaut M., Luczkowiak J., Vives C., Labiod N., Bally I., Lasala F., Grimoire Y., Fenel D., Sattin S., Thielens N. (2021). DC/L-SIGN recognition of spike glycoprotein promotes SARS-CoV-2 trans-infection and can be inhibited by a glycomimetic antagonist. PLoS Pathog..

[bib34] Turonova B., Sikora M., Schurmann C., Hagen W.J.H., Welsch S., Blanc F.E.C., von Bulow S., Gecht M., Bagola K., Horner C. (2020). In situ structural analysis of SARS-CoV-2 spike reveals flexibility mediated by three hinges. Science.

[bib35] Wang S.F., Huang J.C., Lee Y.M., Liu S.J., Chan Y.J., Chau Y.P., Chong P., Chen Y.M. (2008). DC-SIGN mediates avian H5N1 influenza virus infection in cis and in trans. Biochem.Biophys. Res. Commun..

[bib36] Watanabe Y., Bowden T.A., Wilson I.A., Crispin M. (2019). Exploitation of glycosylation in enveloped virus pathobiology. Biochim.Biophys. Acta Gen. Subj..

[bib37] Watanabe Y., Allen J.D., Wrapp D., McLellan J.S., Crispin M. (2020). Site-specific glycan analysis of the SARS-CoV-2 spike. Science.

[bib38] Xia S., Liu M., Wang C., Xu W., Lan Q., Feng S., Qi F., Bao L., Du L., Liu S. (2020). Inhibition of SARS-CoV-2 (previously 2019-nCoV) infection by a highly potent pan-coronavirus fusion inhibitor targeting its spike protein that harbors a high capacity to mediate membrane fusion. Cell Res..

[bib39] Yao H., Song Y., Chen Y., Wu N., Xu J., Sun C., Zhang J., Weng T., Zhang Z., Wu Z. (2020). Molecular architecture of the SARS-CoV-2 virus. Cell.

[bib40] Zhao P., Praissman J.L., Grant O.C., Cai Y., Xiao T., Rosenbalm K.E., Aoki K., Kellman B.P., Bridger R., Barouch D.H. (2020). Virus-receptor interactions of glycosylated SARS-CoV-2 spike and human ACE2 receptor. Cell Host Microbe.

[bib41] Zheng Z., Monteil V.M., Maurer-Stroh S., Yew C.W., Leong C., Mohd-Ismail N.K., Arularasu S.C., Chow V.T.K., Lin R.T.P., Mirazimi A. (2020). Monoclonal antibodies for the S2 subunit of spike of SARS-CoV-1 cross-react with the newly-emerged SARS-CoV-2. Euro Surveill..

[bib42] Zou X., Chen K., Zou J., Han P., Hao J., Han Z. (2020). Single-cell RNA-seq data analysis on the receptor ACE2 expression reveals the potential risk of different human organs vulnerable to 2019-nCoV infection. Front. Med..

