# Supplementary material for: Epigenetic glycosylation of SARS-CoV-2 impact viral infection through DC&L-SIGN receptors
Source: iScience. 2021 Nov 11;24(12):103426. doi: 10.1016/j.isci.2021.103426 (PMC8582233; doi:10.1016/j.isci.2021.103426)

iScience, Volume *24*

Supplemental information

Epigenetic glycosylation of SARS-CoV-2 impact viral infection through DC&L-SIGN receptors

Lei Guo, Yan Liang, Heng Li, Huiwen Zheng, Zening Yang, Yanli Chen, Xin Zhao, Jing Li, Binxiang Li, Haijing Shi, Ming Sun, and Longding Liu

Figure S1. Expression efficiency of ACE2, DC-SIGN, and L-SIGN in Vero, MLE-12, HepG-2, and 16HBE cells by lentiviral transduction. Expression of ACE2, DC-SIGN, and L-SIGN proteins was detected by immunofluorescence using anti-hACE2, hDC-SIGN, and hL-SIGN antibodies together with Alexa Fluor 647-conjugated secondary antibodies, respectively. Images were obtained under confocal microscopy.

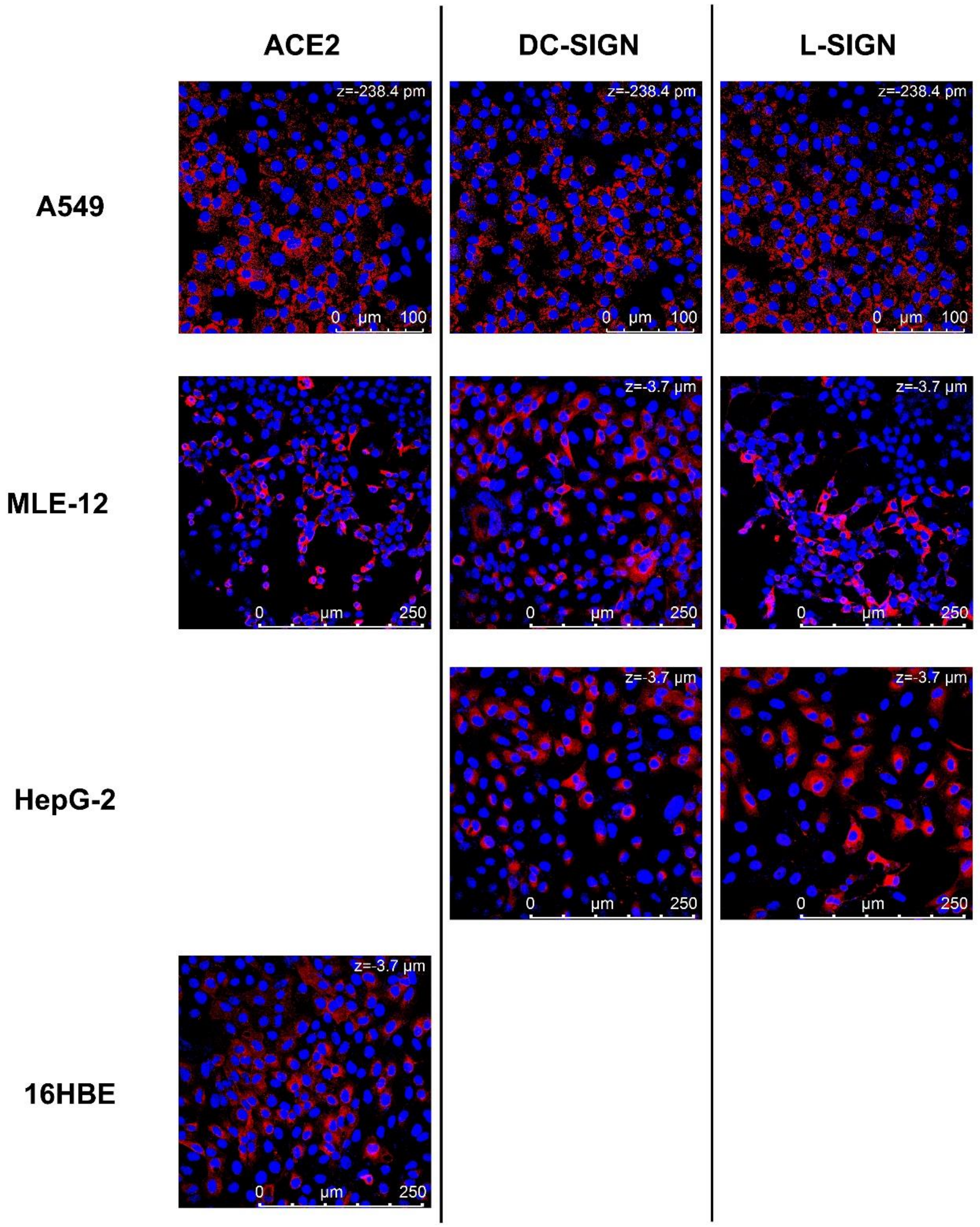

Figure S2. SARS-CoV-2 infection through different receptors. SARS-CoV-2 infection, replication, and proliferation through A549 cells stably expressing ACE2, DC-SIGN, or the L-SIGN receptor by lentiviral transduction (from the top down). A549 cells transduced with empty lentiviral particles were used as a negative control (NC). Left panels, viral loads of the infected cells were determined based on the number of viral envelope (E) gene RNA copies detected by qRT-PCR at the indicated h.p.i.; middle panels, virus-infected cells were visualized using anti-viral nucleocapsid protein antibody by confocal microscopy at 24 h.p.i.; Right panels, viral titers from the culture supernatants of the infected cells were determined using a $CCID_{50}$ assay at the indicated h.p.i.. The error bars represent the standard deviation from four repeats.

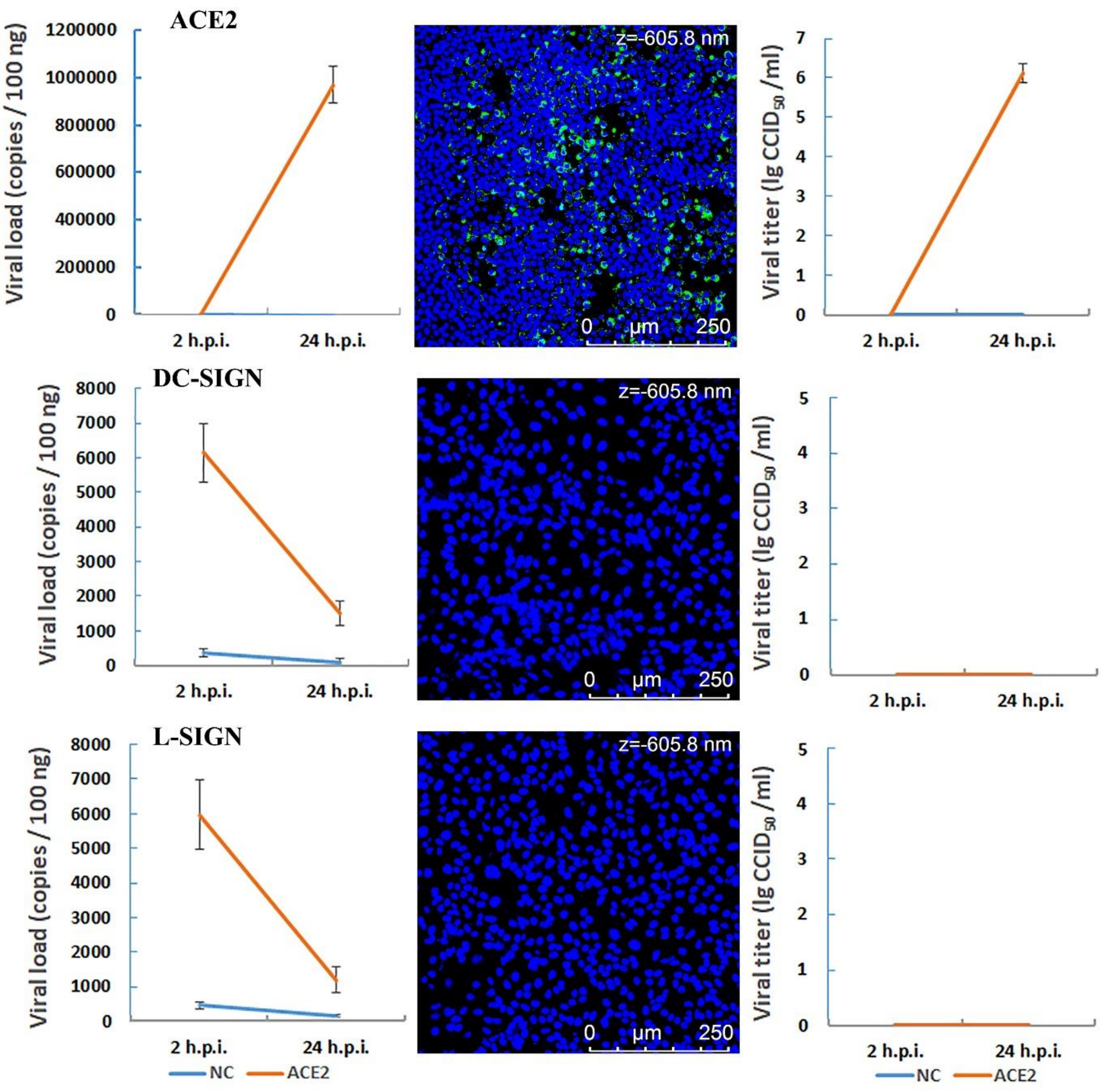

Figure S3. Expression levels of DC-SIGN (CD209) in human peripheral blood monocytes and induced MDDCs by flow cytometry.

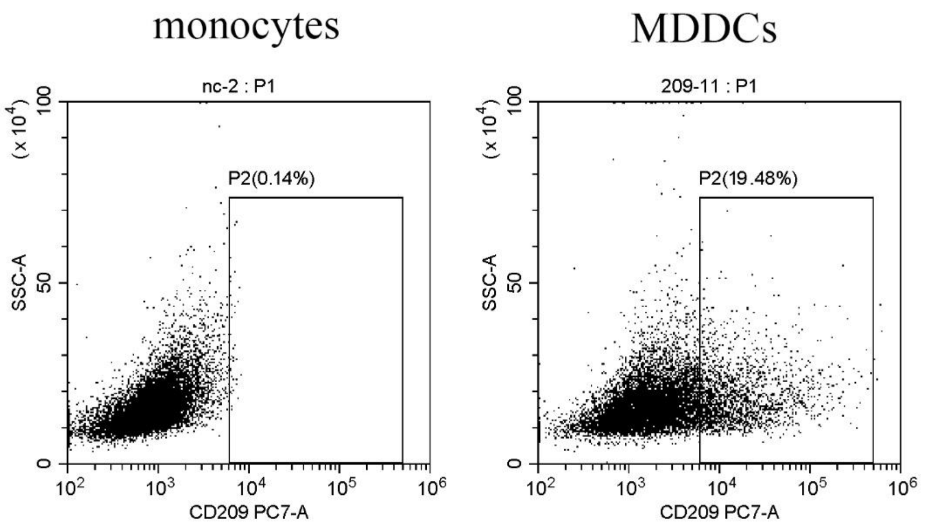

Figure S4. Expression of ACE2 protein in HepG-2 cells, HEpG-2 cells transduced with empty lentiviral particles (NC), and HEpG-2 cells transduced with DC-SIGN or L-SIGN by western blot detection.

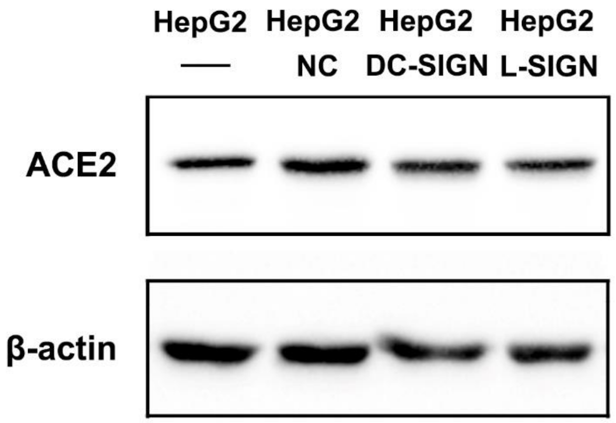

Figure S5. Amino acid sequence alignment of S protein from virions of different cell origins.

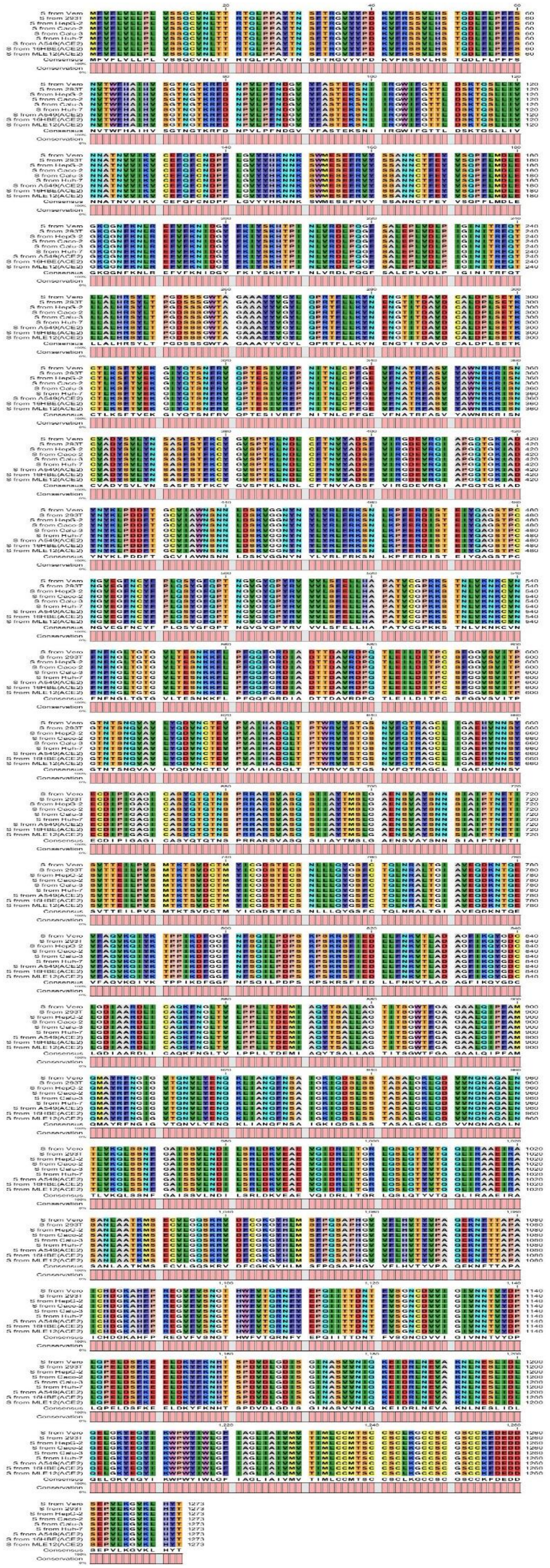

Figure S6. SDS-PAGE analysis of the IP virion samples produced from different host cells (Coomassie blue staining).

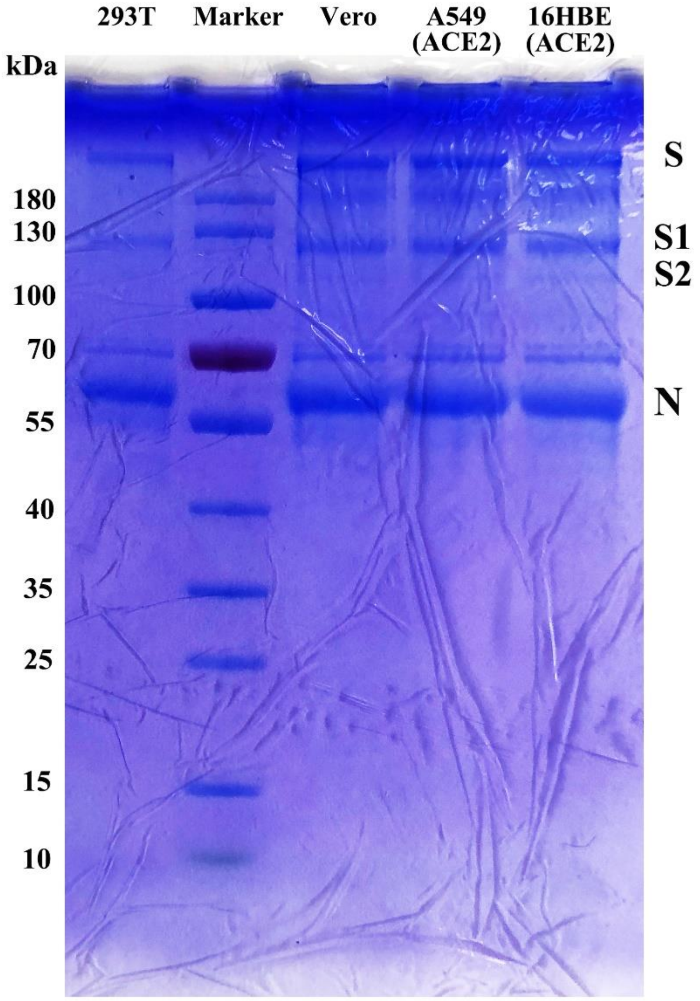

Supplement: Document S1. Figures S1–S6 [file mmc1.pdf]
